# Use of Apology Strategies in Emails by Chinese Learners of English: Evidence Based on Naturally Occurring Data

**DOI:** 10.3389/fpsyg.2021.782613

**Published:** 2022-02-10

**Authors:** Ying Chen, Qi Lu, Yuxuanjing Wei

**Affiliations:** College of Foreign Languages, Ocean University of China, Qingdao, China

**Keywords:** second language pragmatics, apology strategies, strategy combinations, Chinese EFL learners, naturally occurring data

## Abstract

Using a data set of 30 authentic institutional emails written by Chinese college students to their native English teacher, this article investigates the frequency and combinations of apology strategies used by English as a Foreign Language (EFL) learners in natural contexts. Drawing on the coding framework adapted from previous studies, this article carries out a fine-grained analysis of apology behaviors of Chinese EFL learners when they offended their teacher for various reasons. Results revealed that the most frequently used strategy was illustrative force indicating devices (IFIDs), and “IFIDs + taking on responsibility” ranked the most frequent combination. Among IFID sub-strategies, *an expression of regret* had the highest frequency. In addition, a new strategy—*request for a chance to repair*—was identified, which was used by Chinese learners to show their respectful and pious attitude when a more serious offense was made to their teacher. Findings from the study indicate that Chinese EFL learners tend to use culture-specific apology strategies in academic contexts. This study has pedagogical implications for EFL pragmatics instruction in Chinese contexts and also second language pragmatics instruction tailored for native Chinese learners in English-speaking countries.

## Introduction

Apology is one of the most ubiquitous and frequent speech acts in public discourse and social interactions and has long generated a considerable amount of interest over the last 50 years (Goffman, 1971; Cohen and Olshtain, 1981, 1985; Blum-Kulka and Olshtain, 1984; Trosborg, 1987; Suszczyriska, 1999; Dalmau and Hortènsia, 2007; Shakki et al., 2020; Derakhshan and Malmir, 2021). This article takes the apology definition of Holmes (1990, p. 159) as a working definition, which states that “An apology is a speech act addressed to B’s face-needs and intended to remedy an offense for which A takes responsibility, and thus to restore equilibrium between A and B (where A is the apologizer, and B is the person offended).” Apology often begins with a sense of guilt. It shows the regret of the offender about the negative impact inflicted on the victim. By admitting an offense or misdeed, the apologizer damages his or her positive face (Derakhshan and Eslami-Rasekh, 2015; Wang and McGlone, 2020). Therefore, an apology is recognized as a face-threatening act (Brown and Levinson, 1987) and also as a challenging learning target. Language learners who do not know how to make apologies might have problems in their social life and intimate relationship construction, whereas an appropriate apology could save the face of the victim and rebuild harmonious relations (Derakhshan et al., 2021). As a result, it is of paramount significance to investigate how second language (L2) learners make apologies when offending someone. Unfortunately, a close look at the current literature shows that most studies on apology production of L2 learners (Afghari, 2007; Bella, 2014; Liu and Ren, 2016; Al Masaeed et al., 2018; Beeching, 2019; Chang and Ren, 2020) used elicited data rather than real-life performances of learners. Due to the hypothetical situation provided when eliciting apology data by either discourse-completion tasks (DCTs) or role plays, participants lack cognition of the consequences of their behaviors, and correspondingly, their reactions lack authenticity (Malmir and Taji, 2021). Therefore, it is still in question to what extent the elicited data can reflect apology behaviors of L2 learners in natural contexts.

Previous email apology research predominately investigated the apologies of native speakers using authentic data (e.g., Hatipoğlu, 2004; Davies et al., 2007; Claudel, 2015), but employed elicited data when participants were L2 learners (e.g., Liu and Ren, 2016; Barón and Ortega, 2018). To the best of the knowledge of the authors, there has been no published research on apology strategies of L2 learners using authentic email data. Therefore, this article intends to contribute to the field by examining the frequency and combination of apology strategies used by English as a Foreign Language (EFL) learners in natural email communications with their native English teacher.

This article consists of six sections. The following section introduces the research background, notes the significance of the study and the overall structure, and reviews previous research on email apologies, other forms of authentic apologies, and apologies of L2 learners. The methodology of the study is then discussed, and the following section highlights the main findings. The results are discussed in light of the relevant literature, and the final section summarizes the major findings and points out the limitations of this study and also possible directions for future research.

## Theoretical Background

### Previous Research on Email Apologies

The DCT is a predominated way for researchers to collect data on written apologies, compared to which the use of email has received less attention (e.g., Hatipoğlu, 2004; Davies et al., 2007; Chejnova, 2021). However, with daily work and life being closely integrated in the Internet era, email has increasingly replaced traditional letters and become an important means of communication (Hatipoğlu, 2004). The use of institutional emails between teachers and students in this article is a common example.

Based on 126 emails by British English speakers, Hatipoğlu (2004) was among the first to investigate whether email apologies possessed characteristics of spoken or written language. The results indicated that email apologies shared commonalities with apologies in spoken language in that pronouns were used oftentimes, and email apology was similar to the apology in written language in that intensifiers were mostly employed in a majority of cases. In contrast, email apologies contained certain new qualities, which allowed language users to tell it from other registers. Moreover, the author also found that compared with the other sub-strategies of illustrative force indicating devices (IFIDs), a request for forgiveness was seldom used in the corpus. This study supported the paper trail paradox adopted from Baron (1998) that email writing followed more rules in written language than in spoken language.

Collecting 100 emails sent by English undergraduate students, Davies et al. (2007) analyzed the type and sequence of apologies by native learners. Findings revealed that in addition to apologies, there were usually some other speech acts that served as the main motivation, and apologies were just side acts. Students employed apologies to be politer, to “pay debts” or “gain credits,” thus paving way for the main acts. In addition, the authors classified apology based on three temporal factors (past, present, and future) and two spatial factors (THIS and THAT). Finally, this study captured a new strategy, but-justification, which referred to “I may have done something for which I need to apologize, but I’m doing other things that make me a good student/person” (p. 57), unlike accounts in that it did not mitigate blame.

Another study that explored institutional apologies in higher education was Chejnova (2021), which analyzed strategies employed in 200 email apologies of Czech students. Results showed that IFIDs were the most frequent single strategy, and participants preferred to use a combination of IFIDs + accounts and justifications + acknowledgment of responsibility.

It should be noted that the aforementioned three studies all explored email apologies from the perspective of the native speaker. There were also studies on apology emails of L2 learners, but using elicited data. Employing two discourse production tasks, Liu and Ren (2016) investigated email apologies used by two proficiency groups of Chinese EFL learners in two equal-status scenarios (writing to apologize to their foreign peers). Perception analysis showed that both groups had a similar understanding of the severity of offense and social distance in two scenarios. Production data revealed that lower proficiency learners tended to produce longer apologies than their peers. Moreover, compared with the advanced group, students with lower L2 proficiency preferred IFIDs and taking on responsibility.

Barón and Ortega (2018) designed a scenario to allow Catalan/Spanish EFL learners and English native speakers to write request and apology emails. They divided both learners and native speakers into younger and older groups and analyzed their pragmatic moves. Findings revealed a gap between younger learners and older learners, as well as younger learners and native speakers. Older learners apologized less than their foreign peers. In cases where they did consider it necessary to apologize, they chose to provide accounts or explanations.

A review of email apology literature showed that while research on native speakers tended to use authentic emails, research on L2 learners inclusively based their analysis on elicited data. There is of great value to explore apology acts of L2 learners in natural contexts.

### Previous Research on Naturally Occurring Apologies

After surveying 246 empirical studies published from 1979 to 2017, Nguyen (2019) found that only 53 studies (out of 217 pragmatic production studies) used naturalistic data. Authentic data are preferable in pragmatic research because they best reveal real language use (Bardovi-Harlig, 2010). Nevertheless, research based on the analysis of naturally occurring apologies is relatively moderate (Robinson, 2004; Kampf and Blum-Kulka, 2007; Chamanigi and Zareipur, 2010; Shariati and Chamani, 2010; Drew and Hepburn, 2016; Heritage and Raymond, 2016; Heritage et al., 2019), especially considering that apology is a high-risk speech act and that no real-life consequences are involved in these imagined situations that are used to elicit data.

Several studies have used naturalistic data to explore how language users apologize. Holmes (1990) investigated the use of both apology strategies and strategy patterns in a corpus of New Zealand English. Analysis unveiled that nearly a half of apology exchanges involved just one single strategy, almost always an explicit apology, and an explicit apology with an explanation or account formed the most frequent strategy combination selected. On the one hand, Holmes reckoned that the use of strategy combinations necessarily led to a “weightier” apology, appropriate for more serious offenses. On the other hand, results showed that a simple explicit apology was frequently considered adequate by New Zealand English native speakers.

Shariati and Chamani (2010) generated a corpus of 500 apology exchanges in standard spoken Persian through ethnographic observation in 1 year. They analyzed the frequency, combination, and sequential position of apology strategies and found that IFID was used most frequently by Persian speakers, whereas IFID plus acknowledgment of responsibility formed the most common combination. A related study by Chamanigi and Zareipur (2010) compared the use of apologies and the offenses that motivated apologies in Persian and British English. Results showed that accidents were the most common source of apologies in Persian, while hearing offenses led to the highest rate of apologies in British English. Furthermore, the exclusive use of explicit apology was the most frequent strategy in British English, and explicit apology with minimization formed the most used strategy combination in Persian.

With an over-4.5-million-tokens corpus of Ghanaian parliamentary apologies from 2005 to 2018, Sarfo-Kantankah (2021) investigated the apologetic expression and sincerity exhibited in them. Findings revealed “sorry,” “withdraw,” and “apology” as the most frequent expressions. Besides, sincerity was shown through the use of a variety of apology strategies, including acknowledgment of the offense, expression of remorse or regret, acceptance of responsibility, offer of repair, and promise of forbearance.

Although these studies shed light on the use of apology strategies by native speakers from various cultures, there is a lack of investigation into how L2 learners use apology strategies and strategy combinations, let alone that of Chinese EFL learners. Besides, none of these studies explored written apologies. Given the stark differences between oral and written language processing methods, it is worth exploring whether there are any differences in apology strategies employed in emails and oral conditions.

### Previous Research on Apologies of Chinese English as a Foreign Language Learners

This study analyzed the apology behaviors of Chinese EFL learners in emails. As a limited number of research in this area have been conducted, how Chinese EFL learners apologize in other registers would provide a useful reference.

Using cartoon oral production tasks, Rose (2000) investigated requests, apologies, and compliment responses produced by three groups of primary school English learners, with their Cantonese native speaker peers as a baseline. Analysis of apology data of learners found that all three groups overwhelmingly used IFIDs, followed by taking on responsibility. However, no group exhibited the ability to change apology strategy effectively with regard to situations, neither pragmatic transfer occurred from Cantonese, a Southern Chinese dialect, to English.

Chang (2010) examined apologies elicited from a DCT, which composed of eight scenarios, produced by four proficiency groups of participants, ranging from preliminary school students to undergraduate students. Results suggested that as L2 proficiency increased, the range of apology strategies used also increased. Some strategies were used only by advanced learners. Specifically, “I’m sorry/sorry” occurred in an early stage of second language acquisition, while “request for forgiveness” emerged at a relatively late stage. Concerning the frequency of strategies, IFIDs were the most frequently used strategies. Among the three sub-strategies of IFIDs, expressing regret occurred most often while a request for forgiveness occurred least times.

Apparently, the apology behaviors of Chinese EFL learners were largely under-researched. Therefore, this article aims to investigate apologies made by Chinese EFL learners to their native English teacher through emails. Given that “apologizing properly may not just be a matter of expressing regret through a single word” (Margutti et al., 2016, p. 64), this article focuses on the use of both single apology strategies and strategy combinations by Chinese EFL learners. To this end, the two research questions to be addressed in this study are as follows:

1) What types of apology strategies do Chinese EFL learners employ when they apologize to their native English teacher in email?
2) In what combinations do Chinese EFL learners use apology strategies?

## Materials and Methods

### Participants

Twenty-seven Chinese undergraduate English major students at a university in east China participated in this study, including 22 female participants and 5 male participants. They varied from freshmen to senior students (age range: 18–22 years). All participants took different courses of the same English teacher and offended the English teacher for various reasons. As a large portion of off-class communication between the foreign teacher and students is done through emails, they chose to apologize by email. One participant sent four emails to the native English teacher for different reasons, and the other 26 participants each sent one email to apologize to their English teacher.

### Data Collection

This study explored apology production of Chinese EFL learners in written English. According to the study by Nguyen (2019, p. 196), compared with other kinds of naturally occurring data, “computer-mediated communication provides a convenient source of data because the data are digitally recorded and immediately available for analysis.” Therefore, computer-mediated communication (CMC) data were collected. Email, as a common type of CMC, was chosen because email is increasingly becoming an important communication tool, and apology in email is not uncommon. Thirty authentic emails written by 27 Chinese college students to apologize to their English teacher who is an English native speaker were collected. This native speaker is a very “demanding” teacher who has stringent requirements for his classes. He received many kinds of apology emails from different students in different classes. The third author of this article is a student of the English teacher. The authors contacted the participants through the English teacher. All participants agreed to contribute their emails for research purposes, and they were informed that their names or other personal information would be excluded from the data analysis and that there were no known risks connected with participating in this project.

### Coding Scheme and Data Analysis

For the coding of these emails, a researcher first conducted the data coding alone, and then, another researcher who has experience in the coding of speech act data checked the data coding. The two researchers discussed the discrepancy until they reached an agreement. The coding scheme used in this study was adopted from the framework of strategies reported by Olshtain and Cohen (1983) and Trosborg (1987) (see Table 1). The apology strategy *request for the chance to repair* was added to the scheme after coding the emails used in this study. It was politer than the strategy *offer of repair* since it asks the opinion of the victim.

**TABLE 1:** Coding scheme for apology strategy.

**1.**	**Illocutionary force indicating devices (IFIDs)**
	a. An expression of regret, e.g., I’m sorry.
	b. An offer of apology, e.g., I apologize.
	c. A request for forgiveness, e.g., Excuse me/Forgive me/Pardon me.
**2.**	**Explanation or account, e.g., There was a traffic jam.**
**3.**	**Acknowledgment of responsibility**
	a. Explicit self-blame, e.g., It is my fault/my mistake.
	b. Lack of intent, e.g., I didn’t mean it.
	c. Expression of self-deficiency, e.g., I was confused/l didn’t see you/I forgot.
	d. Expression of embarrassment, e.g., I feel awful about it.
	e. Justify hearer, e.g., You’re right to be angry.
	f. Self-dispraise, e.g., I’m such a dimwit!
**4.**	**Denial of responsibility**
	a. Explicit denial of responsibility, e.g., It wasn’t my fault.
	b. Pretend to be offended, e.g., I’m the one to be offended.
	c. Blame the hearer, e.g., It’s your own fault.
**5.**	**Offer of repair, e.g., I’ll pay for the damage.**
**6.**	**Promise of forbearance, e.g., It won’t happen again.**
**7.**	**Request for the chance to repair, e.g., Hope you can give me a chance for once.**

For the analysis of emails, the authors first clarified some basic information about emails. The email dates varied over a 34-month period (from April 2018 to December 2020). The situations in these emails were consistent in one contextual factor, that is, the relative power between the apologizer (student, in low power) and the victim (teacher, in high power). Besides, since the offense types were typical in college situations, they were not categorized based on the framework provided by Deutschmann (2003). Instead, the researchers made a more fine-grained classification of offense types for this study. The reasons for writing apology emails and the number of emails accordingly can be seen in Table 2.

**TABLE 2:** Summary of offensive scenarios.

Offense types	Frequency	Percentage (%)
Quitting class	7	23.33
Sending wrong homework	6	20.00
Failing to meet the deadline	4	13.33
Absence	4	13.33
Late reply	2	6.67
Using electronic devices in class	1	3.33
Butting the teacher	1	3.33
Doing bad presentation	1	3.33
Changing the presentation topic	1	3.33
Failing to find study materials	1	3.33
Changing email address	1	3.33
Failing the exam	1	3.33
Total	30	100.0

From Table 2, it can be seen clearly that dropping class was the most common (23.33%) offense to be remedied with an apology, followed by sending wrong homework (20%). The aforementioned offense types together with failing to meet the deadline and absence accounted for 70% of the whole database. All the offense types are familiar to college students, and they can represent some of the offenses in real-life situations. Further analysis concerning apology strategies used in the corpus will be displayed in the next section.

## Results

In this section, the frequencies and combinations of apology strategies used by Chinese EFL learners are summarized in great depth.

### Apology Strategies

For the 30 emails analyzed in this study, a total of 142 apology strategies used by 27 participants were recognized. In all the emails, participants used more than one apology strategy. Table 3 illustrates the frequency and percentage of different strategies.

**TABLE 3:** Apology strategies used in the corpus.

Apology strategies	Subcategories	Frequency	Percentage (%)
IFIDs	An expression of regret	36	25.175
	An offer of apology	9	6.294
	A request for forgiveness	6	4.196
	Total	51	35.664
Taking on responsibility	Expression of self-deficiency	24	16.901
	Expression of embarrassment	7	4.930
	Lack of intent	5	3.521
	Explicit self-blame	4	2.817
	Self-dispraise	3	2.113
	Justification for hearer	1	0.699
	Total	44	30.769
Denial of responsibility	Explicit denial	6	4.196
	Pretend to be offended	6	4.196
	Blame the hearer	2	1.399
	Total	13	9.091
Explanation or account		14	9.790
Offer of repair		11	7.692
Request for a chance to repair		6	4.196
Promise of forbearance		4	2.797
Total		143	100.0

Table 3 demonstrates that apology strategies used by Chinese EFL learners were far from being evenly distributed. IFIDs were the most frequently used apology strategy (51 times occurrences in total, taking up more than one-third of all strategies used). In all the apology exchanges, there was at least one IFID used. The data suggested that IFIDs were realized through various forms, including *an expression of regret* (e.g., “I’m sorry to bother you,” “I’m indeed very sorry for my absence,” and “I’m sincerely sorry for what I have done”), *an offer of apology* (“I am writing to apologize for my decision that I cannot take your class this semester,” “Once again, I apologize for any inconvenience caused”), and *a request of forgiveness* (“I would be deeply grateful if you can understand me and accept my apologies,” “I beg for your forgiveness”). *An expression of regret* was the most common sub-strategies, taking up 70% of the whole category. In addition, in their expression of regret, participants preferred to add intensifiers, such as “so,” “indeed,” “very,” “really,” “sincerely,” to strengthen their tone of regret, thus making their apologies more acceptable.

*Taking on responsibility* was the second frequent strategy used by Chinese EFL learners. A series of sub-strategies were identified in the corpus, among which *expression of self-deficiency* (e.g., “Perhaps I misunderstand you,” “I remembered the wrong time”) with 24 occurrences occupied more than a half of the whole. Besides, participants had a preference for the expression of embarrassment (e.g., “At that time, I was too shocked to speak, for I’ve never come across this kind of situation,” “I feel very sad and pity”) and lack of intent (e.g., “It is his fault that he didn’t make sure he was sending the right thing, but he didn’t know what went wrong”). Only one participant justified the hearer in the apology.

In contrast, the strategy *denial of responsibility* was also found in the data (9.1%), with a variety of sub-strategies, including *explicit denial* (e.g., “It was not totally our fault,” “To be honest, I don’t think I did anything wrong and I tried my best”), *pretending to be offended* (e.g., “I think it unfair to me to some degree,” “We believe we are one of the most hard-working students in the course. But those, who did work hard and failed in the midterm, could have a chance to make up their score. It was unfair”), and *blaming the hearer* (e.g., “If you checked it out, we would at least have time to find the reason and send you our video. Your reply seemed to already received it,” “I think you got the wrong document”).

Moreover, the other two strategies that participants used more than ten times were: *explanation or account* (e.g., “Since I’m having a driver test now, I couldn’t catch the car to school right now,” “The mail notification was not opened”), and *offer of repair* (e.g., “If you still hope that I can pick one of the four previous topics for a speech, I will re-write an outline to you,” “I will send you the video with my roommate’s email”). *A promise of forbearance* (e.g., “I promise not to violate your rules during the class such as playing mobile phone”) was the least used strategy in this corpus (2.8%).

Finally, the researchers recognized a new strategy—*request for a chance to repair* (e.g., “Sincerely hope you could give us the chance to make up the mid-term”) with an occurrence of 6 times from different participants. Instead of coming up with a method to repair the loss or suffering of the victim, the apologizer only asked the victim to give him or her a chance to repair it. It was the victim to decide how to compensate for the offense. It showed more respect for the victim, thus was politer than offering repairs directly.

### Combinations of Apology Strategies

As mentioned earlier, since all the apology exchanges in the data involved certain combinations of strategies, a mere description of single apology strategies was not enough. Table 4 shows all the strategy combinations used in 30 emails.

**TABLE 4:** Strategy combinations in the corpus.

Strategy combination	Frequency
AA	1
AACC	1
ABBAGCAD	1
ABBCA	1
ABC	1
ABCADA	1
ABCCA	1
ABCD	1
ABD	1
AC	1
ACA	1
ACA	1
ACAG	1
ACC	1
ACCA	1
ACCAAA	1
ACCADA	1
ACCCACAACEA	1
ACE	1
ADDCB	1
AGGA	1
BFAFFFD	1
CA	1
CACC	1
CBCCA	1
CCCAE	1
CCCCAAA	1
DA	1
FFBBFFCFA	1
FFF	1

*A, IFID; B, an explanation or account; C, take on responsibility; D, an offer of repair; E, a promise of forbearance; F, denial of responsibility; G, request for a chance to repair.*

It can be seen from Table 4 that most participants employed more than three apology strategies in an individual email. The range of strategy numbers used in a single email was quite large. To be more specific, there were at least two strategies and at most eleven strategies. Furthermore, 30 emails had 30 different strategy combinations. However, some commonalities could still be found. Based on their number of occurrences, Table 5 summarizes those combinations.

**TABLE 5:** Combination patterns of apology strategies.

Patterns	Frequency	Percentage (%)
AC + X × N	10	28.33
AB + X × N	7	23.33
C × N + A + X × N	4	13.33
F × N + X × N	3	10.00
AA + X × N	2	6.67
A + X × N	2	6.67
Others	2	6.67
Total	30	100

*A, IFID; B, an explanation or account; C, take on responsibility; F, denial of responsibility; X, any other strategies; N, natural number.*

Results presented in Table 5 showed that the combination of IFID + taking on responsibility + any other strategies was the most commonly used strategy combination (10 times). IFID + an explanation or account + any other strategies ranked the second (7 times). The remaining combinations were in an even distribution, occurring no more than 5 times each. Examples (1–6) show refusals of patterns summarized by Table 5.


(1) AC+X×N

I am so sorry for what I said just now. I realize how disrespectful and rude I was. I know it’s my responsibility that I don’t prepare it….


(2) AB+X×N

I would like to sincerely apologize for not attending the last few classes as I am preparing for a make-up exam of politics. I will attend this class every Friday from next week. I will pay attention to the class, I promise not to violate your rules during the class such as playing mobile phone. This letter is to explain to you why I failed to attend the previous classes and to tell you about my course plan. I hope I didn’t miss too much of the course.


(3) C×N+A+X×N

I found later that I really miss the deadline. I don’t know how to explain that to you – “I remembered the wrong time.” Sometimes I am timid and reasonless. I was so scared at the time, so I dared not add any words. I’m really sorry.


(4) F×N+X×N

Although I think it is unfair to me to some degree, I think I have no choice but to accept your decision. As a team, we have divided our work. It was my responsibility to cut the video and R’s to send it. And I did make verification after he sent it. To be honest, I don’t think I did anything wrong and I tried my best.


(5) AA+X×N

I’m sorry to bother you. I’m writing to apologize for the mistake I made in using the timer for my classmate … I have been in your class for one year and I know exactly and obey the rules that we shouldn’t use any electronic devices for notetaking, entertainment, and contact. But perhaps I misunderstood you, for I’m sure that we can’t use timers to do our own presentations, but still can use it for my classmates….


(6) A+X×N

I’m sorry that I can’t attend the class this Wednesday because I need to participate in the volunteer activities….

## Discussion

This study investigated the use of apology strategies and combinations of apology strategies by Chinese EFL learners in their emails written to an English teacher. Results revealed that the most used single apology strategy was explicit IFID, with *an expression of regret* having the highest frequency among any other sub-strategies, while explicit IFID + taking on responsibility + any other strategies formed the most frequent combination of apology strategies.

### Apology Strategies

As mentioned in the previous section, IFIDs were the most frequently used apology strategy. This was also reported in previous studies such as Holmes (1990), Rose (2000), Chang (2010), Chamanigi and Zareipur (2010), Shariati and Chamani (2010), Liu and Ren (2016), and Chejnova (2021). Of the three sub-strategies, the highest occurrence belonged to *an expression of regret*, similar to Chang (2010), while a request for forgiveness was used least frequently which is in agreement with Hatipoğlu (2004) and Chang (2010). IFIDs are explicit apologies, whereas the others are implicit apologies (Malmir and Taji, 2021). The result suggests that Chinese EFL learners, like other language users, have a preference for explicit apologies since they can clearly express their intent.

Moreover, Table 3 shows that *denial of responsibility* was the fourth most used apology strategy in this study, which was quite different from many previous studies which drew on either elicited data (Olshtain and Cohen, 1990; Bella, 2014; Prachanant, 2016) or authentic data (Holmes, 1990; Shariati and Chamani, 2010), in that this strategy was not found in any of these studies. In a DCT or role-play situation, participants are usually asked to perform an apology, which means that little room is given to refuse responsibility. Moreover, without real-life consequences, participants usually imagine their responses in an idealistic way, which may not be consistent with what they will do in real situations. Furthermore, the data used in this study were different from other authentic-data-based studies in two aspects: first, it was in the written form than in the spoken form. Thus, an apologizer had enough time for consideration before he or she wrote apology emails. The apologizer might ponder whether it was his or her fault, and there was a good chance that he or she would deny the responsibility if the answer was no. Second, all remedial exchanges in the data were between students and the native English teacher. Certain interests, such as their score, of students were involved. There was a consequence if they chose to take on the responsibility. At the same time, it should be noted that *denial of responsibility* is also one strategy that largely depends on the situation. Therefore, these two differences might help explain why *denial of responsibility* was not found in other naturally occurring-data-based studies. What is more, the most frequently used sub-strategy of *denial of responsibility* in this study was *explicit denial*, while the least used sub-strategy was *to blame the receiver*. It indicates that although the participants did not admit their fault in the denial-of-responsibility situations, most of them would not blame the receiver.

Furthermore, this article also identifies the *request for a chance to repair* as an apology strategy, which was not observed in any other previous studies. *Request for a chance to repair* is politer than *offer of repair* since it is the victim other than the apologizer to decide how to compensate for the offense. In this way, more respect is shown for the victim. As a result, this strategy is appropriate when a more serious offense was made to a person of higher status. In the following example, a student wrote an email to apologize for sending the wrong video, which was an assignment by the English teacher. At the end of the email, she asked for a chance to repair:

“Sincerely hope you could give us the chance to make up the mid-term. I am sorry!”

As a student, the apologizer was not in the right place to offer a repair directly for sending the wrong video. Therefore, a request for a chance to repair was adopted to make the receiver, the English teacher, in this case, feel more comfortable, more respected, and less imposed. Also, the apologizer seemed sincere and humble through the use of this strategy.

Finally, unlike Hatipoğlu (2004), which found that email apologies shared commonalities with other forms of written language in that neither of them employed substantial intensifiers, the apology emails in this study contained a relatively large number of intensifiers to stress the feelings of apologizers. A possible explanation is that in the corpus of Hatipoğlu (2004), quite some emails were “professional,” that is, the offenders were apologizing either for an offense that was “required” by their post in college or apologizing on behalf of a group of people. By contrast, all the emails collected in this study were from undergraduate students to their teacher, who was of a higher social status. The students showed more respect and their pious attitude through the use of intensifiers.

### Combination of Apology Strategies

Different from Holmes (1990) which stated that nearly half of the apologies involved only one apology strategy, all the emails in this corpus contained at least two apology strategies. One possible reason might be that although the corpus of Holmes contained written data, most of her apology exchanges were in spoken language. When apologizing in spoken language, the speakers are going through online processing and need to act in a short period of time. As a result, chances are that they might end up with a single strategy in their apologies. The corpus of this study, on the contrary, was composed of written apologies. Writers can weigh and consider what kind of apology strategy is appropriate and can help to mitigate the offenses they made to the other party. As combined strategies lead to a weightier apology (Holmes, 1990), Chinese EFL learners used different strategy combinations when writing to their native English teacher to apologize for their misdeeds.

Regarding the frequency of strategy combinations, the results of this study suggested that the combination of IFID + taking on responsibility + any other strategies was the most commonly used strategy combination. This finding is in line with Shariati and Chamani (2010) and Liu and Ren (2016), but at odds with those reported by Holmes (1990) and Chejnova (2021). Holmes (1990) observed that an explicit apology with an account or explanation was the most common combination used by New Zealanders. Similar to Holmes (1990), Chejnova (2021) also found that IFIDs + accounts and justifications + acknowledge of responsibility formed the most frequent combination. This difference may reflect the influences of cultural norms and values on the choice of apology strategies of language users, that is, certain cultural transfer exists in the apology behaviors of Chinese EFL learners in emails. According to Assandri and Meisterernst (2019, p. 15), “Chinese is one of the few languages in which an early philosophical and logical system developed independently of an influence from any systems in Indo-European languages.” The values cherished by and the spiritual pursuit of the Chinese people are quite different from those of western countries such as New Zealand and the Czechia. Westerners prefer explaining after an explicit apology, which seems to build a distance between the interlocutors. Moreover, the explanation or account for the offensive behaviors of an individual is often a kind of external force majeure, so as to excuse the apologizer from responsibility and reduce the dissatisfactory feelings of the other party. Hence, offering an explanation is in essence the self-justification of the apologizer. In contrast, Chinese people are more likely to take on the responsibility for the offense to save the face of the victim. This comes down to Chinese culture. Confucianism, Daoism, and Buddhism are popularly perceived as sanjiao (literally “three teachings”) that constitute the threefold system of Chinese culture (Shi et al., 2019). Confucius believed that a Junzi (Man of Virtue) must have a sense of responsibility, which is the core value of Confucianism. Daoism also stressed the social responsibility of loving people and things based on social roles. Under such influences, Chinese people have stressed the importance of responsibility through the generations. A man of responsibility is oftentimes equated to a role model in Chinese culture who is supposed to be emulated in the community or at the national scale. Growing up and being cultivated in such culture, the sense of responsibility becomes the prominent attribute valued by Chinese people and is frequently regarded as the sign of a mature person. Such social norms and cultural identity have a great impact on the pragmatic performance of the learners (Malmir and Derakhshan, 2020a,b). At the same time, “face culture” is an important part of Chinese traditional culture, which permeates the behaviors of Chinese people and greatly affects the daily social life of Chinese people. The emergence of face culture has a lot to do with traditional Confucian culture and the human society of China. Confucianism generally emphasizes the use of etiquette and the preciousness of harmony. Therefore, Chinese people would like to bear the responsibility of the offense to maintain harmonious interpersonal relationships.

## Conclusion

This study investigated the apology behaviors of Chinese EFL learners by collecting and analyzing naturally occurring data, that is, apology emails of Chinese EFL learners to their native English teacher. The findings showed that IFIDs were the most frequently used strategies and the most common combination was an IFID with taking on responsibility. This study also identified a new strategy, *request for a chance to repair*, which was used when a more serious offense is made to a person of higher status. In terms of differences in apology strategies and strategy combinations reported in previous studies, we inferred that they might result from data collection methods, email types, and cultural norms. Based on authentic data, the analysis provides insights into the use of apology strategies and strategy combinations by Chinese EFL learners. We argue that attention should be paid to social and cultural factors, and analysis should be made on the differences between the target language and mother tongue, so as to truly learn the target language well and use it appropriately in different contexts.

This study is limited in several ways. First and foremost, a challenge involved in authentic data is that “social situations in data vary greatly, rendering it difficult to compare pragmatic behaviors across participants or time points” (Nguyen, 2019, p. 197). Future research should advisably classify email types in terms of social distance, power, and other contextual information. Second, the data set used in this study is very small, and only 30 authentic emails were collected which unavoidably limits the generalizability of the findings. Future research can build a large corpus of authentic emails of L2 learners to verify the findings yielded. Furthermore, this study only explored the apology behaviors of Chinese EFL learners in real-life EFL classroom contexts without reference to how they make apologies in similar L1 contexts and how native English speakers apologize in such contexts. Apology performances made by both native Chinese and English speakers need, therefore, to be explored in future studies to provide baselines.

## Data Availability Statement

The original contributions presented in the study are included in the article/supplementary material, further inquiries can be directed to the corresponding author/s.

## Ethics Statement

The studies involving human participants were reviewed and approved by the Ethics Committee at Ocean University of China. The patients/participants provided their written informed consent to participate in this study.

## Author Contributions

YC contributed to conception and design of the study. YW organized the database and wrote the first draft of the manuscript. YW and QL performed the statistical analysis. YC and QL wrote sections of the manuscript. All authors contributed to manuscript revision, read, and approved the submitted version.

## Conflict of Interest

The authors declare that the research was conducted in the absence of any commercial or financial relationships that could be construed as a potential conflict of interest.

## Publisher’s Note

All claims expressed in this article are solely those of the authors and do not necessarily represent those of their affiliated organizations, or those of the publisher, the editors and the reviewers. Any product that may be evaluated in this article, or claim that may be made by its manufacturer, is not guaranteed or endorsed by the publisher.
